# Systematic metabolic engineering for trans-aconitic acid production in *Aspergillus niger*

**DOI:** 10.1016/j.synbio.2026.10.007

**Published:** 2026-11-02

**Authors:** Song Wang, Jiaying He, Wenhao Li, Chenlu Cui, Jiao Liu, Hao Liu

**Affiliations:** Key Laboratory of Industrial Fermentation Microbiology, Ministry of Education, Tianjin Key Laboratory of Industrial Microbiology, College of Biotechnology, Tianjin University of Science and Technology, No. 9, 13th Avenue, Tianjin Economic-Technological Development Area (TEDA), Tianjin, 300457, PR China

**Keywords:** *Aspergillus niger*, Aconitic acid, Metabolic engineering, Push-pull strategy

## Abstract

Trans-aconitate (TAA) is a valuable bio-based chemical for nematicides and polymer synthesis. We repurposed the industrial citrate producer *Aspergillus niger* for efficient TAA biosynthesis. Heterologous AexA enabled extracellular aconitate export. However, overexpressing aconitases from various sources, targeted to the cytoplasm or mitochondria, failed to increase titers. Although mitochondria proved the optimal site for cis-aconitate accumulation, the bottleneck likely persisted due to thermodynamic constraints. To overcome this, we designed a push–pull strategy: a pull module using mitochondrial MttA to export cis-aconitate to the cytosol, where TbrA isomerizes it to TAA, coupled with a push module enhancing cytosolic acetyl-CoA via the phosphoketolase pathway. Multicopy tbrA expression was critical. The final strain produced 34.30 g/L total aconitate (>90% TAA) in a 5-L bioreactor within 6 days. This work highlights a compartmentalized push–pull strategy exploiting mitochondrial-cytosolic organization to convert a citrate chassis into a high-yield TAA cell factory.

## 1 Introduction

Aconitic acid (prop-1-ene-1,2,3-tricarboxylic acid), an unsaturated tricarboxylic acid first isolated from plants of the genus *Aconitum* (*Aconitum napellus*) [1], [2], exists as two geometric isomers: cis-aconitic acid (CAA) and trans-aconitic acid (TAA) [3]. Functionally distinct, CAA serves as a transient metabolic intermediate in the aconitase-catalyzed interconversion of citrate and isocitrate within the tricarboxylic acid (TCA) cycle. In contrast, TAA is biosynthesized via two distinct pathways: (1) spontaneous isomerization of CAA under physiological conditions, which confers enhanced environmental stability compared to CAA; or (2) enzymatic isomerization mediated by aconitate-Δ-isomerase (e.g., TbrA), enabling directional enrichment of TAA with high specificity [4], [5], [6], [7]. TAA exhibits dual agricultural applications: potent nematicidal activity against plant-parasitic nematodes and robust antifeedant effects against *Nilaparvata lugens* at environmentally relevant concentrations, offering sustainable alternatives to synthetic pesticides in integrated pest management [8], [9]. These properties position TAA as a key molecule for advancing sustainable crop protection amid tightening regulatory constraints on conventional pesticides.

Microbial production has emerged as the most viable route for sustainable TAA manufacturing, with different microbial chassis offering distinct advantages and inherent limitations. While significant progress has been made in engineering diverse hosts for aconitic acid production, substantial bottlenecks remain that are specific to each system. In *Aspergillus terreus*, deletion of *cadA* (encoding cis-aconitate decarboxylase) blocked the itaconic acid pathway, enabling initial CAA accumulation (10.8 g/L in 5 days); subsequent heterologous expression of aconitate-Δ-isomerase TbrA from *Bacillus thuringiensis*, combined with fermentation optimization, achieved 60 g/L TAA in a 20 m^3^ industrial bioreactor [10], though concerns over slow growth, morphological complexity, and potential mycotoxin production remain. Similarly, *A. pseudoterreus* with *cadA* knockout produced 10 g/L aconitic acid, and overexpression of the MFS-family transporter *AexA* boosted titers to 50 g/L, highlighting the critical role of efficient export [11]. In *Yarrowia lipolytica*, redirection of carbon flux via knockout of triacylglycerol synthesis (*DGA1/2*, *LRO1*, *ARE1*) and sterol esterification (*SE*) genes, followed by disruption of *isocitrate lyase* (*ICL1/2*) and cytosolic *NADP*^*+*^ -dependent isocitrate dehydrogenase (*IDP*), increased CAA yield 2.93-fold; however, the essentiality of mitochondrial *NAD*^*+*^ -dependent IDH (*IDH1/2*) constrained further flux redirection [12]. By contrast, *Escherichia coli*—despite CRISPRi-mediated attenuation of *pyruvate kinase* and *isocitrate dehydrogenase* to enhance precursor availability and intermediate retention—achieved only 0.62 g/L due to CAA's transient metabolic role and negligible extracellular secretion [13]. More recently, systematic elimination of mixed-acid fermentation pathways combined with anaplerotic flux enhancement enabled *E. coli* to produce 37.32 g/L TAA with a yield of 0.76 g/g, demonstrating that prokaryotic hosts can achieve high TAA yield through upstream push strategies [14]. *Aspergillus niger*, a GRAS organism with industrial-scale citric acid production (∼200 g/L) [15], [16], [17], offers an ideal endogenous citrate supply for CAA biosynthesis; yet current engineering efforts, including overexpression of CadA from *A. terreus*, the mitochondrial transporter MttA, and aconitase mAcoA, have yielded only 9.8 g/L after 240 h [18], [19], indicating substantial untapped potential and the need for a more integrated strategy to overcome transport, metabolic competition, and precursor limitations unique to this host.

The low yield of aconitic acid in *A. niger* is notable given its robust citrate-producing capacity. We identified three interrelated limitations that likely account for this discrepancy. First, transmembrane transport of aconitate is incomplete. While heterologous overexpression of the mitochondrial tricarboxylate carrier MttA has been employed to enhance CAA export from mitochondria into the cytosol, the subsequent step—efficient secretion of CAA (or TAA) from the cytosol to the extracellular space—remains unaddressed. Notably, the MFS-family exporter AexA, which was shown to boost aconitate titers in *A. pseudoterreus* [11]*,* has not been implemented in *A. niger*. Moreover, the native plasma membrane transport system for aconitic acid in this host is still undefined, leaving intracellular accumulation likely and hindering both product recovery and accurate metabolic analysis [20]. Second, metabolic flux is inherently unfavorable for CAA accumulation: endogenous aconitase rapidly converts CAA to isocitrate within the TCA cycle, and the reaction equilibrium strongly favors citrate (citrate:CAA:isocitrate ≈ 91:3:6 at pH 7.4, 25 °C), constraining net CAA formation in the absence of product removal [21]. Third, downstream isomerization has not been engineered. Critically, heterologous aconitate-Δ-isomerase (e.g., TbrA from *B. thuringiensis* or Adi1 from *Ustilago maydis*), which catalyzes the directional conversion of CAA to TAA and thereby provides an enzymatic pull that can thermodynamically drive mitochondrial CAA export, has not been functionally implemented in *A. niger* [22], [23]. Without such a pull mechanism, CAA remains trapped in a reversible metabolic loop between citrate and isocitrate, limiting both titer and productivity.

To address these limitations, an integrated metabolic engineering strategy was implemented in *A. niger* based on a push–pull framework and multi-gene coordination, as shown in Fig. 1. The approach comprised the following key components: (i) heterologous expression of the aconitate exporter AexA from *A. pseudoterreus* to enable extracellular secretion; (ii) overexpression of aconitase to enhance conversion of citrate to CAA; (iii) overexpression of the mitochondrial tricarboxylate transporter MttA to facilitate CAA export from mitochondria; (iv) co-expression of phosphoketolase (PK) and phosphotransacetylase (PTA) to strengthen acetyl-CoA supply; (v) introduction of the heterologous aconitate-Δ-isomerase to catalyze directional isomerization of CAA to TAA; and (vi) multi-copy integration of pathway genes combined with optimization of fermentation medium and bioreactor conditions. This engineered strain achieved a significantly improved aconitic acid titer compared to previously reported *A. niger* strains, providing a promising platform for further strain development and process scale-up.

**Fig. 1:**
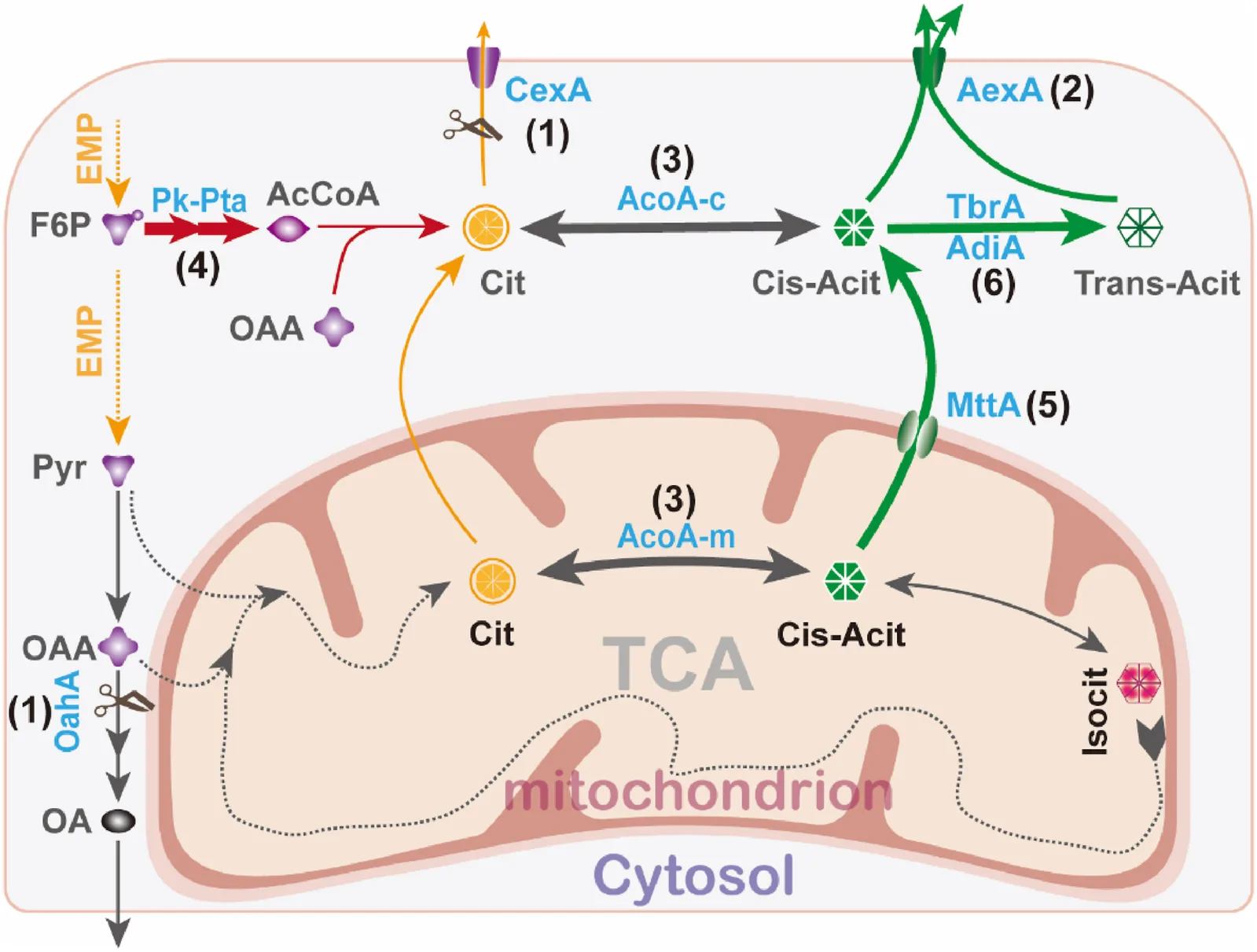
Modular design of the aconitic acid biosynthetic pathway. (1) Knockout OahA: oxaloacetic hydrolase; Knockout CexA: Citrate transporter. (2) Overexpression AexA: aconitate membrane transporter. (3) Overexpression AcoA^c^: Cytosolic aconitase; Overexpression AcoA^m^: mitochondrion aconitase. (4) Overexpression Pk-Pta: Phosphoketolase-phosphate transacetylase. (5) Overexpression MttA: tricarboxylate mitochondrial transporter. (6) Overexpression TbrA/AdiA: aconitate-Δ-isomerase.

## 2 Materials and methods

### 2.1 Strains and culture conditions

The *A. niger* strains utilized in this study are detailed in Table S1. Strain S1075 (Δ*oahA*, Δ*cexA*), a derivative of *A. niger* ATCC 1015, was used as the parental strain for all subsequent genetic modifications [15]. *Escherichia coli* JM109 was used for plasmid construction and propagation and cultured in Luria-Bertani (LB) medium at 37 °C. Spore preparation, transformant screening, and phenotypic analysis of mutants were carried out on potato dextrose agar (PDA), following previously described protocols [24]. Plate cultures were incubated at 28 °C, whereas liquid fermentations were performed at 35 °C. Where necessary, media were supplemented with 250 μg/mL hygromycin B or 100 μg/mL kanamycin for selection during strain construction and plasmid maintenance [24]. The original AA fermentation medium used in shake flask experiments was adapted from protocols originally developed for itaconic acid production by *A. niger* [25], with some modification. The initial basal fermentation medium contained (per liter): 100.0 g glucose, 2.0 μg biotin, 2.0 g (NH_4_)_2_SO_4_, 50.0 mg sodium citrate dihydrate, 100.0 mg KH_2_PO_4_, 4.0 mg MgSO_4_·7H_2_O, 2.0 mg CaCl_2_·2H_2_O, 20.0 μg citric acid monohydrate, 20.0 μg ZnSO_4_·7H_2_O, 4.0 μg Fe(NH_4_)_2_(SO_4_)_2_·6H_2_O, 1.0 μg CuSO_4_·5H_2_O, 0.2 μg H_3_BO_3_, 0.2 mg Na_2_MoO_4_, and 1 M MES, adjusted to pH 6.5. This original medium served as the starting point for the optimization experiments presented in Section 3.5.

### 2.2 Construction of plasmids

All plasmids utilized in this study are listed in Table S1, and the corresponding primers are provided in Table S1 of the Supporting Information. Plasmid construction was performed using the ClonExpress II One Step Cloning Kit (C112-01/02) supplied by Nanjing Vazyme Biotech Co., Ltd. (Nanjing, Jiangsu Province, China). The sequences of *aexA*, *Ate*-*acoA*, *mttA*, *pk*, *pta*, *adiA* and *tbrA* were synthesized by GENEWIZ (Suzhou) Co., Ltd., whereas the sequences of *Ang*-*acoA* was amplified from the cDNA of strain S1075, the sequences of *SC*-*acoA* was amplified from the cDNA of *S. cerevisiae*.

The plasmids for expressing *aexA*, *acoA*, *mttA*, *pk*, *pta*, *tbrA* and *adiA* were constructed following the same strategy as described in a previous study, using plasmid pLH454 as the backbone. For instance, the *aexA* overexpression plasmid (pLH2156) was generated by inserting the *aexA* coding sequence (CDS) downstream of the glyceraldehyde-3-phosphate dehydrogenase promoter (P*gpdA*) in pLH454 [26]. PCR amplification was performed using the synthesized gene as the template and the primer pair 1/2. The resulting PCR product was subsequently ligated into pLH454 digested with *Bam*HI and *Eco*RI, yielding the recombinant plasmid pLH2156. Following the same methodology, the *Ang*-*acoA*^*C*^ expression plasmid pLH2184, *Ang*-*acoA*^*M*^ expression plasmid pLH2185, *Ate*-*acoA*^*C*^ expression plasmid pLH2186, *Ate*-*acoA*^*M*^ expression plasmid pLH2187, *Sc*-*acoA*^*C*^ expression plasmid pLH2188, *Sc*-*acoA*^*M*^ expression plasmid pLH2189, *mttA* expression plasmid pLH2331, *pk*-*pta* co-expression plasmid pLH2076, *tbrA* expression plasmid pLH2342, and *adiA* expression plasmid pLH2343 were constructed.

### 2.3 Construction of strains

A series of recombinant *A. niger* strains were constructed using PEG-mediated protoplast transformation method, combined with a Cre-*lox*P system for excision of the *hph* resistance marker [24]. All plasmids were introduced into recipient strains via protoplast transformation method. After transformation, *hph* marker genes flanked by *lox*P sites were removed by culturing the transformants on potato dextrose agar (PDA) plates containing doxycycline (30 μg/mL), followed by selection of hygromycin B-sensitive colonies and confirmation by PCR using primers *hph*-F/*hph*-R.

For gene overexpression, plasmids carrying target coding sequences under the control of the P*gpdA* and P*pkiA* were used. Transformants were selected on PDA plates containing hygromycin B (250 μg/mL) at 28 °C for 5 days. Colonies resistant to hygromycin B were further verified on PDA plates with 250 μg/mL hygromycin B, and correct cassette integration was confirmed by PCR.

Strain construction steps were as follows: The overexpression plasmid pLH2156 was first introduced into *A. niger* S1075 to obtain S4060, followed by *hph* excision to generate S4096. Strain S4152, S4154, S4156, S4158, S4160 and S4162 were obtained by S4096 through overexpressing *acoA* genes from different sources that are localized in different organelles, respectively. The *hph* marker was then removed from S4154 to generate S4216. S4216 was used to construct the co-overexpression *pk*-*pta* mutant via transformation with pLH2076 generate S4370, followed by *hph* excision to obtain S4373. S4373 was then transformed with pLH2331 to obtain S4420, from which S4423 was derived. Using strain S4423 as the parental strain, *adiA* and *tbrA* were overexpressed respectively, yielding strains S4427 and S4430. The *hph* marker was then removed from S4430 to generate S4492. S4492 was used as the starting strain to introduce the second copy of three key genes—*aexA*, *mttA*, and *tbrA*, generating strains S4686, S4688, and S4690, respectively. The *hph* marker was then removed from S4690 to generate S5120. Eventually, using strain S5120 as the parental strain, the strain S5126 with three copies of the *tbrA* gene was obtained.

All expression cassettes were stably integrated into the chromosome of *A. niger*, and no episomal plasmids were used for the production strains. Therefore, the engineered pathways are maintained without the need for continuous antibiotic selection.

### 2.4 Transcription analysis of the key genes

An appropriate volume of RNA was taken and reverse-transcribed into complementary DNA (cDNA) according to the instructions of the reverse transcriptase, followed by reverse transcription-polymerase chain reaction (RT-PCR). For quantitative real-time PCR (qRT-PCR), cDNA was first synthesized via reverse transcription in accordance with the protocol of the qRT-PCR kit. Subsequently, the reaction system was prepared using the TB Green Premix Ex *Taq*II method, and quantitative real-time PCR reactions were performed on the Applied Biosystems StepOne™ Real-Time PCR System Thermal Cycling Block. The actin-encoding gene (*act*) of *A. niger* was used as the reference gene, and all reactions were set up in triplicate. The threshold cycle (Ct) value for the amplification of each target gene was normalized to the Ct value of the reference gene (*act*). The relative changes in gene transcription levels between the selected strains and the parental strain were calculated using the 2^−ΔΔCt^ method.

### 2.5 Shaking flask fermentation of *A. niger*

The fermentation and sampling protocol was adapted from a previously described method [25]. Noculate 2.0 × 10^6^ spores/mL of *A. niger* into 250-mL Erlenmeyer flasks containing 50 mL of aconitic acid fermentation medium. Subsequently, conduct shake flask cultivation under conditions set at 35 °C temperature and 250 rpm speed. Measure the evaporation of water in the shake flasks daily and replenish it promptly. Sample for aconitic acid content or residual sugar detection after 6 days of fermentation.

### 2.6 Bioreactor fermentation of *A. niger*

The fermentation protocol was adapted from previously reported methods [26]. Seed cultures were prepared by inoculating the engineered *A. niger* into 250 mL Erlenmeyer flasks containing 50 mL of the AA fermentation medium without MES, and incubated at 35 °C and 250 rpm for 24 h. The resulting seed cultures were then transferred into 2.7 L of fermentation media in 5 L bioreactors and cultivated at 35 °C for 7 days. During fermentation, different medium formulations were evaluated to investigate their effects on AA production. The stirring speed was set at 250 rpm, and the aeration rate was maintained at 3 vvm (volume of air per volume of medium per minute). Fermentation broth was collected every 24 h. The supernatant was used for AA quantification, and the mycelium was harvested using pre-weighed microfiber filters and dried at 80 °C to determine dry cell weight. In all bioreactor runs, the agitation speed and aeration rate were maintained constant from inoculation to harvest (0–144 h). No dynamic or phase-specific control was applied in this study.

### 2.7 Analytical method

The CAA, TAA and CA were determined using high-performance liquid chromatography (HPLC) (Agilent 1260 system) equipped with a refractive index detector (Agilent G1362A). The analysis was performed using a Bio-Rad Aminex HPX-87H column with 5 mM H_2_SO_4_ as the mobile phase. The column temperature was maintained at 65 °C, and the flow rate was set at 0.6 mL/min. UV detector wavelength is 210 nm.

Glucose concentration was measured using an SBA-40E biosensor analyzer (Biology Institute of Shandong Academy of Sciences, China) [24]. Sucrose concentrations were determined using HPLC Agilent 1260 system equipped with a variable wavelength detector. Separation was carried out on a COSMOSIL 5C18-MS-II column (4.6 mm × 250 mm), with a mobile phase consisting of methanol and 0.02 mol/L K_2_HPO_4_ in a ratio of 18:82 (v/v). The flow rate was maintained at 1.0 mL/min, the column temperature at 25 °C. Detection was performed at 273 nm.

### 2.8 Statistical analysis

All fermentation-related data, including CAA, TAA and CA concentration, Biomass, and residual sugar levels, were obtained from three biological replicates and are presented as mean ± standard deviation. Gene transcription levels of *acoA*, *aexA*, *mtta* and *tbrA* were measured from five biological replicates. Statistical analyses were performed using GraphPad Prism software. One-way or two-way analysis of variance (ANOVA) was applied as appropriate to assess statistical significance.

## 3 Results

### 3.1 Installing an aconitate exporter relieves feedback inhibition and reveals a downstream bottleneck

Although *A. niger* possesses the intrinsic capacity to synthesize aconitic acid, its native production is minimal. The parental strain S1075 was derived from a citrate hyperproducer. In this strain, the major citrate exporter gene *cexA* had been deleted to reduce background citrate secretion and to redirect carbon flux toward aconitate biosynthesis. After 6 days of shake-flask fermentation, S1075 produced only 0.15 g/L CAA and 0.44 g/L TAA, yielding a total aconitic acid titer of 0.58 g/L (Fig. 2A and B). Notably, TAA accounted for 75.2% of total AA, indicating that spontaneous isomerization of CAA to TAA occurs during cultivation even in the absence of a heterologous isomerase. This confirms that *A. niger* can endogenously produce and secrete aconitic acid, albeit at very low levels.

**Fig. 2:**
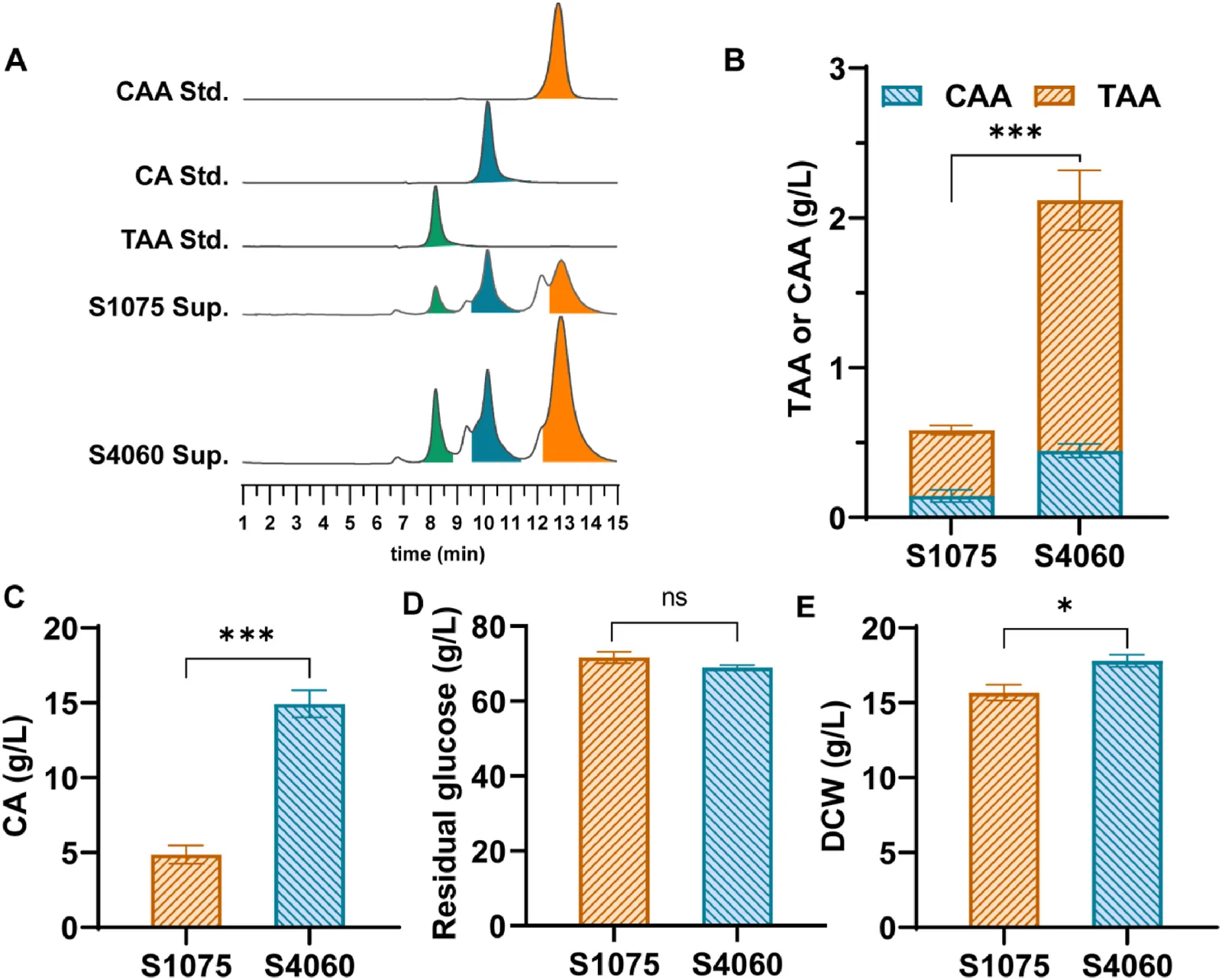
Effect of the AexA transporter on AA production in 6-day shake-flask cultures. (A) HPLC chromatograms of fermentation broths from strains S1075 and S4060, and organic acid standards (CAA, TAA, and CA). (B) AA titer, (C) CA titer, (D) residual glucose, and (E) DCW. All engineered strains were derived from S1075 (*ΔoahA, ΔcexA*). S4060 (S1075 + OE-*aexA*). Abbreviations: TAA, cis-aconitic acid; CAA, trans-aconitic acid; AA, total aconitic acid; CA, citric acid; DCW, dry cell weight.

Membrane transporters are indispensable for organic acid production in *A. niger* [27], [28], [29]. Previous studies have mostly focused on modifying intracellular biosynthetic pathways and optimizing fermentation processes to improve organic acid yields. However, without an efficient transport system, organic acids accumulate inside the cell. This causes feedback inhibition of metabolic pathways, exerts toxicity on cells, and ultimately reduces production efficiency. The molecular mechanisms governing aconitate transport remain poorly characterized, and no functionally characterized aconitate exporter has been identified in *A. niger*. Given the critical role of efficient secretion in organic acid production, we sought to functionally install a heterologous transporter with demonstrated aconitate efflux activity. Magnuson et al. [11], reported that the protein encoded by *aexA* in *A. pseudoterreus* exhibits aconitate transport activity, with *aexA* overexpression leading to a 5-fold increase in aconitic acid titer relative to the wild-type strain. Although homologous transporter candidates may exist in *A. niger*, no aconitate transporter with experimentally validated activity has been identified in this host. Therefore, we introduced *A. pseudoterreus* AexA, whose aconitate export activity has been functionally characterized, to establish an effective aconitate secretion module in *A. niger*.

The expression cassette *PgpdA::aexA::TtrpC* was integrated into the genome of S1075 (*ΔcexA*), generating the *aexA*-overexpressing strain S4060. Comparative shake-flask fermentations revealed that, despite *cexA* knockout, S1075 still secreted 4.87 g/L citrate after 6 days, confirming residual citrate export through alternative pathways (Fig. 2A and C). In contrast, S4060 exhibited markedly enhanced aconitic acid production: CAA and TAA titers reached 0.45 g/L and 1.67 g/L, respectively, yielding a total AA concentration of 2.12 g/L. These values represent 3.0-fold, 3.8-fold, and 3.7-fold increases over S1075 for CAA, TAA, and total AA, respectively (Fig. 2A and B), and the TAA fraction rose to 78.8%. These results demonstrate that AexA functions as an effective aconitate exporter in *A. niger*. Notably, citrate secretion in S4060 also increased substantially, reaching 14.93 g/L, a 3.1-fold rise compared with S1075 (Fig. 2A and C). Concurrently, residual glucose in S4060 decreased to 69.03 g/L from 71.73 g/L in S1075, and the mass yield of total AA on consumed glucose improved from 0.021 to 0.068 g/g. Dry cell weight increased moderately by 13.6% (Fig. 2D and E). The coordinated enhancement in organic acid production, carbon utilization, and biomass is consistent with the notion that AexA-mediated efflux may help relieve intracellular accumulation of tricarboxylic acids, potentially mitigating feedback inhibition on central metabolism. Direct measurement of intracellular metabolite concentrations would be required to confirm this interpretation.

The concurrent elevation of both citrate and aconitate in the supernatant of S4060 is noteworthy. This co-export phenotype could reflect substrate promiscuity of AexA. For example, AexA might transport citrate or other TCA intermediates in addition to aconitate. Alternatively, AexA-mediated aconitate efflux may relieve intracellular feedback inhibition on citrate synthase. This would enhance citrate biosynthesis, and the excess citrate could then be exported through endogenous carriers still present in the *cexA*-deficient background. These two mechanisms are not mutually exclusive, and the present data do not distinguish between them. *In vitro* transport assays with purified AexA reconstituted into liposomes will be required to definitively establish its substrate spectrum. Regardless of the precise mechanism, this result implies that engineering aconitate secretion via a single transporter can exert broader effects on the TCA cycle metabolic network. AexA thus represents a potential key node for modulating flux distribution within the TCA cycle. This offers a novel strategy for overproducing TCA-derived compounds.

The substantial citrate accumulation in S4060 also points to a downstream bottleneck. Specifically, the aconitase-catalyzed conversion of citrate to CAA appears to limit overall flux. Since S1075 was originally selected for exceptional citrate production, its engineered derivatives likely retain strong carbon flux entering the TCA cycle. However, the downstream enzymatic steps have not yet been optimized to match this flux. Consequently, enhancing the efficiency of citrate-to-CAA conversion remains a key challenge for further improving aconitic acid yields in *A. niger*.

This may require protein engineering of aconitase to shift reaction equilibrium or increase catalytic efficiency, together with optimization of cofactor availability. Looking ahead, selecting or engineering AexA variants with higher specificity for aconitate could further reduce citrate co-production and improve process selectivity.

### 3.2 Aconitase overexpression alone is insufficient without product removal

The preceding section indicated that the conversion of citrate to CAA likely constitutes a bottleneck. To evaluate whether increasing *acoA* gene dosage could alleviate this limitation, six expression constructs were designed. The genes were sourced from *A. niger* (*Ang*), *A. terreus* (*Ate*), and *S. cerevisiae* (*Sc*), with each variant targeted to either the cytoplasm (C) or mitochondria (M). The constructs were individually introduced into strain S4096, a marker-free derivative of S4060 that produced 2.12 g/L total AA and a TAA fraction of 78.8%. The resulting strains were S4152 (*Ang*-*acoA*^C^), S4154 (*Ang*-*acoA*^M^), S4156 (*Ate*-*acoA*^C^), S4158 (*Ate*-*acoA*^M^), S4160 (Sc-*acoA*^C^), and S4162 (*Sc*-*acoA*^M^). Shake-flask fermentations were performed with S4096 as the control, and organic acid profiles were analyzed after 6 days.

Cytoplasmic overexpression consistently reduced total AA production (Fig. 3A). S4152, S4156, and S4160 produced 1.67 g/L, 1.62 g/L, and 1.76 g/L total AA, respectively. These values correspond to decreases of 21%, 24%, and 17% compared with S4096. CAA remained low across these strains (0.35∼0.43 g/L). Citrate levels were slightly higher than that of S4096 (14.93 g/L), ranging from 15.03 to 15.10 g/L. DCW increased to 19.3∼20.7 g/L (Fig. S1A). The TAA/AA ratio stayed near 79%. These results indicate that cytosolic aconitase overexpression does not improve aconitate production and may instead redirect a small portion of carbon away from the pathway. In contrast, mitochondrial overexpression resulted in minimal changes (Fig. 3B). S4154 (*Ang*-*acoA*^M^) produced the highest titer among the six engineered strains: CAA 0.55 g/L, TAA 1.69 g/L, and total AA 2.24 g/L. This represented only a 5.7% increase over S4096. Citrate (14.67 g/L) and residual glucose (78.17 g/L) were not significantly altered, while DCW increased by 9.6% to 19.50 g/L (Fig. S1B). Strains S4158 and S4162 showed even smaller differences relative to S4096. RT-qPCR confirmed that the introduced *acoA* genes were actively transcribed in all strains (Fig. 3C). Endogenous *Ang-acoA* was strongly upregulated in S4152 and S4154, and heterologous *acoA* transcripts were detected in the respective transformants at moderate levels (Fig. 3D). These data confirm that the lack of phenotypic improvement was not due to the absence of transcript accumulation.

**Fig. 3:**
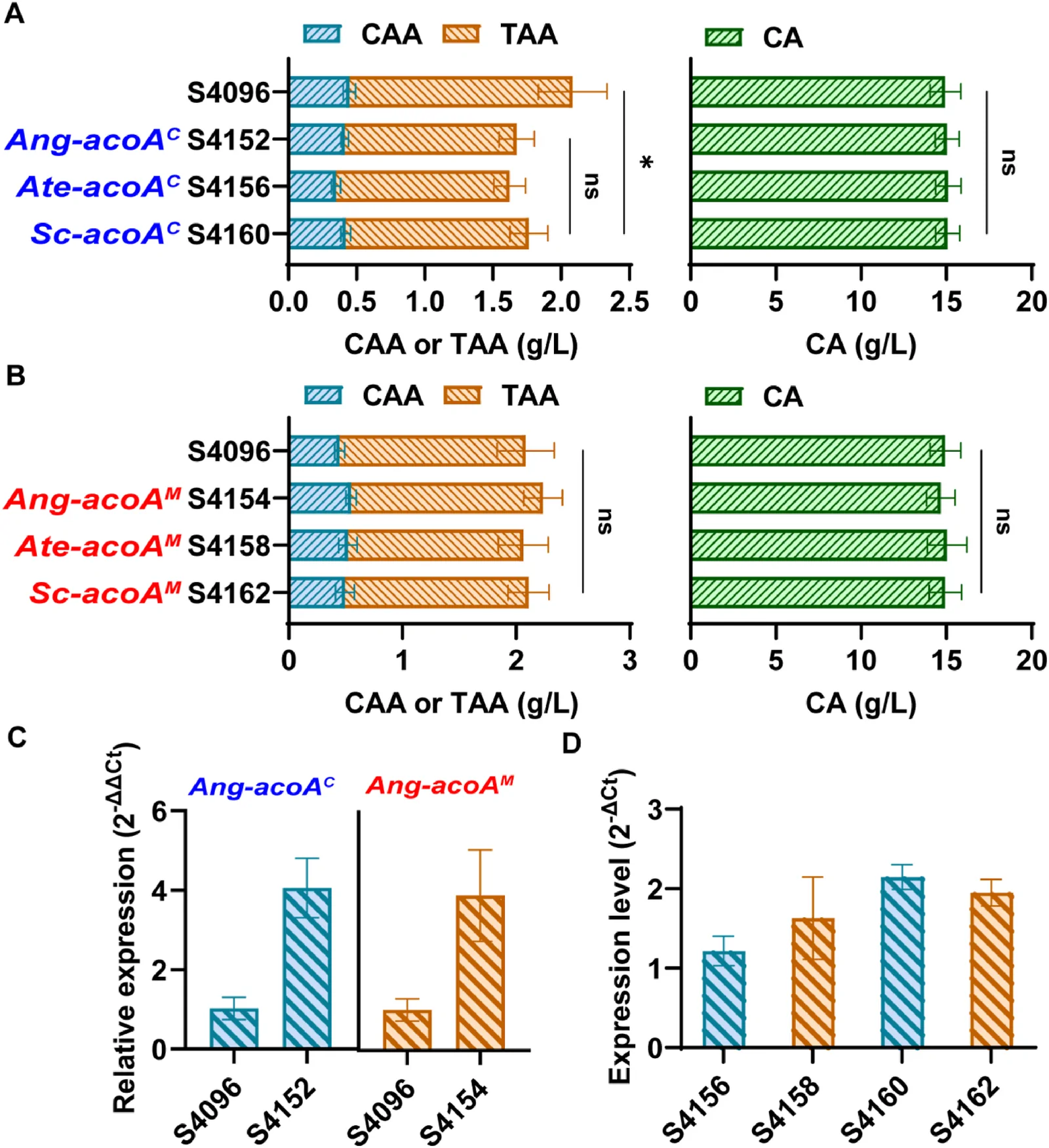
Impact of aconitase overexpression on AA production in 6-day shake-flask cultures. AA and CA titers in engineered strains overexpressing *acoA* localized in the (A) cytoplasm or (B) mitochondria. Relative transcription levels of (C) endogenous and (D) heterologous *acoA* analyzed by qRT-PCR. S4096 (OE-*aexA*), S4152 (S4096 + OE-*Ang-acoA*^C^), S4154 (S4096 + OE-*Ang-acoA*^M^), S4156 (S4096 + OE-*Ate-acoA*^C^), S4158 (S4096 + OE-*Ate-acoA*^M^), S4160 (S4096 + OE-*Sc-acoA*^C^), and S4162 (S4096 + OE-*Sc-acoA*^M^). *Ang-acoA*, *Ate-acoA*, and *Sc-acoA* represent *acoA* genes from *A*. *niger*, *A*. *terreus*, and *Saccharomyces cerevisiae*, respectively. M: mitochondrial localization; C: cytoplasmic localization. Abbreviations: TAA, cis-aconitic acid; CAA, trans-aconitic acid; AA, total aconitic acid; CA, citric acid; DCW, dry cell weight.

The markedly different outcomes between cytoplasmic and mitochondrial expression highlight that subcellular localization is critical for aconitate metabolism. Cytoplasmic overexpression consistently reduced yield. This suggests that the cytosolic microenvironment is unfavorable for net CAA accumulation, possibly due to suboptimal pH, redox conditions, or insufficient cofactor availability. Mitochondrial targeting aligns with the native localization of aconitase. Even so, the improvement was marginal. This indicates that the conversion of citrate to CAA may be not primarily limited by the amount of aconitase protein present. The limited effect of mitochondrial overexpression is consistent with the thermodynamic properties of the aconitase reaction, which strongly favors citrate under physiological conditions. Under standard conditions (pH 7.4, 25 °C), the equilibrium mixture contains approximately 91% citrate, 6% isocitrate, and 3% CAA (BRENDA: EC 4.2.1.3). Without active removal of CAA, the reaction strongly favors citrate, restricting net CAA accumulation. The persistent accumulation of citrate alongside static aconitate titers across all engineered strains is consistent with this thermodynamic interpretation. The small advantage seen in the mitochondrial strains is consistent with the view that mitochondria are the major site of citrate-to-CAA conversion in *A. niger*. This interpretation is in line with previous work showing that co-expression of *acoA* with the mitochondrial CAA transporter *mttA* produced a much larger increase in yield [18]. The underlying principle is that active removal of CAA from its site of synthesis pulls the equilibrium toward the product.

In summary, increasing *acoA* gene dosage is not an effective strategy for improving aconitic acid production in *A. niger*. The bottleneck at this step may be thermodynamic rather than limited by gene transcription. The next engineering steps should therefore focus on pulling CAA away from its site of synthesis. This can be achieved by actively exporting CAA from mitochondria and converting it to a stable product, thereby overcoming the equilibrium constraint.

### 3.3 A push–pull strategy redirects carbon flux from citrate to trans-aconitate

Because aconitase overexpression alone proved insufficient, a compartmentalized push–pull strategy was implemented. The push module aimed to increase cytosolic acetyl-CoA supply by co-overexpressing phosphoketolase (PK) and phosphotransacetylase (PTA) [26]. The pull module combined mitochondrial CAA export via the tricarboxylate transporter MttA with cytoplasmic isomerization to TAA by aconitate-Δ-isomerase.

First, the *pk–pta* dual-expression cassette was integrated into the genome of strain S4216, a marker-free derivative of S4154 that produced 2.18 g/L total AA. The resulting strain S4370 was evaluated in the shake flasks fermentation. After 6 days, S4370 produced 0.56 g/L CAA and 1.81 g/L TAA, yielding a total AA titer of 2.38 g/L. This represented an 8.7% increase over S4216 (Fig. 4A). Citrate accumulation rose significantly to 18.03 g/L, a 21.8% increase compared with S4216 (Fig. 4B). Residual glucose decreased to 63.60 g/L, and DCW increased to 24.70 g/L (Fig. 4C and D). The TAA fraction was 76.1%. These results indicate that enhanced acetyl-CoA supply via the PK–PTA pathway modestly improves aconitate output. However, the concurrent rise in citrate suggests that precursor availability is not the primary bottleneck.

**Fig. 4:**
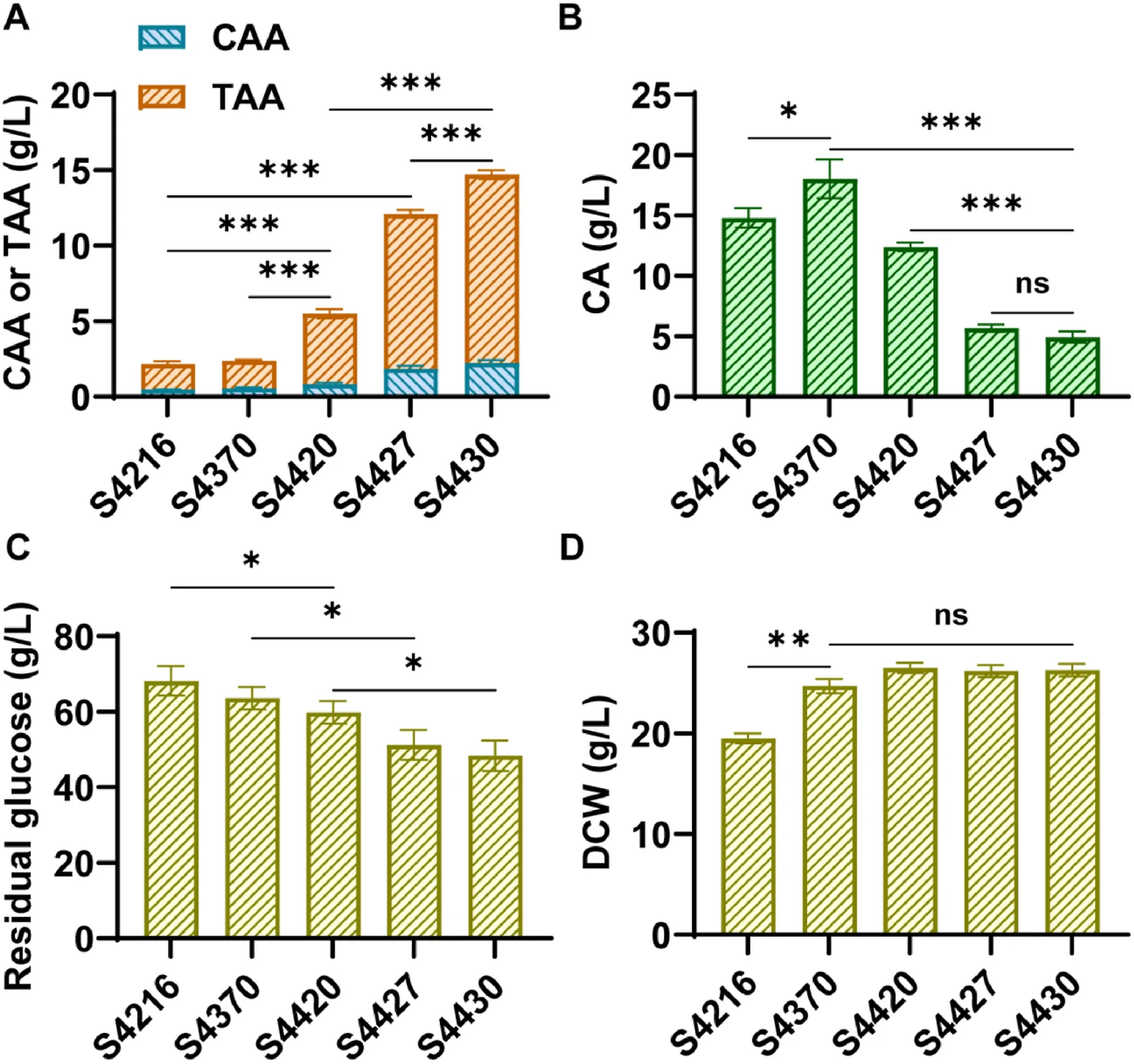
Effect of push–pull strategy on AA production in 6-day shake-flask cultures. (A) AA titer, (B) CA titer, (C) residual glucose, and (D) DCW. S4216 (OE-*aexA*, OE-*Ang-acoA*^M^), S4370 (S4216 + OE-*pk-pta*), S4420 (S4370 + OE-*mttA*), S4427 (S4420 + OE-*adiA*), and S4430 (S4420 + OE-*tbrA*). Abbreviations: TAA, cis-aconitic acid; CAA, trans-aconitic acid; AA, total aconitic acid; CA, citric acid; DCW, dry cell weight.

Next, the mitochondrial tricarboxylate transporter gene *mttA* from *A. terreus* was introduced into strain S4373, a marker-free derivative of S4370. The resulting strain S4420 showed a pronounced shift in product profile. Total AA reached 5.50 g/L, a 2.3-fold increase over S4373. TAA accounted for 84.9% of total AA (Fig. 4A). Citrate dropped sharply to 12.40 g/L, a decrease of 31.2% (Fig. 4B). Residual glucose fell to 59.80 g/L, and DCW rose to 26.50 g/L (Fig. 4C and D). These results are consistent with the role of MttA in facilitating directional export of CAA from mitochondria to the cytoplasm. This export may pull the aconitase reaction forward and reduce citrate accumulation.

Since TAA is the major extracellular form of aconitic acid in *A. niger* fermentations, efficient CAA-to-TAA isomerization was hypothesized to be critical for further yield improvement. No native aconitate-Δ-isomerase has been identified in *A. niger*. Two heterologous candidates were therefore evaluated. The *adiA* gene from *Ustilago maydis* and *tbrA* from *Bacillus thuringiensis* were separately integrated into strain S4423, a derivative of S4420, generating strains S4427 and S4430. Both isomerases strongly enhanced aconitic acid accumulation (Fig. 4A). S4427 produced 12.10 g/L total AA, a 2.2-fold increase over S4423. TAA represented 85.0% of total AA. Citrate decreased to 5.67 g/L (Fig. 4B), a reduction of 54.4%, and residual glucose dropped to 51.20 g/L (Fig. 4C). S4430 performed even better, producing 14.70 g/L total AA, a 2.7-fold increase over S4423 (Fig. 4A). TAA accounted for 84.8% of total AA. Citrate fell to 4.93 g/L (Fig. 4B), a reduction of 60.2%, and residual glucose decreased to 48.33 g/L (Fig. 4C). Biomass remained high in both strains, ranging from 26.2 to 26.4 g/L (Fig. 4D). The high TAA fraction in both strains suggests effective conversion of CAA to TAA after introducing heterologous aconitate isomerases. The superior performance of TbrA led to its selection for subsequent engineering.

Taken together, the integrated push–pull strategy transformed the metabolic profile of the host. Relative to the pre-strategy baseline strain S4096, total AA titer increased approximately 6.9-fold, from 2.12 g/L to 14.70 g/L. Citrate decreased by 67.0%, from 14.93 g/L to 4.93 g/L. Residual glucose declined from 77.90 g/L to 48.33 g/L. These coordinated improvements strongly suggest that mitochondrial CAA export and enhanced cytoplasmic CAA-to-TAA conversion relieved the thermodynamic constraints identified in the preceding section by increasing downstream metabolic pull.

### 3.4 Multicopy trans-isomerase establishes isomerization as the key rate-limiting step

To identify the residual bottleneck in the high-yield background, additional copies of *aexA*, *mttA*, or *tbrA* were introduced into strain S4492. This strain is a derivative of S4430 and produced 14.70 g/L total AA, with a TAA/AA ratio of 84.8% and citrate of 4.93 g/L.

An extra copy of *aexA* was integrated into S4492, generating strain S4686. Shake-flask fermentation showed no significant change in total AA (14.86 g/L) or TAA fraction (84.6%) compared with S4492 (Fig. 5A). Citrate decreased slightly to 4.67 g/L (Fig. 5B). Residual glucose and biomass remained unchanged (Fig. 5C and D). These results indicate that aconitate export capacity is not limited in this background.

**Fig. 5:**
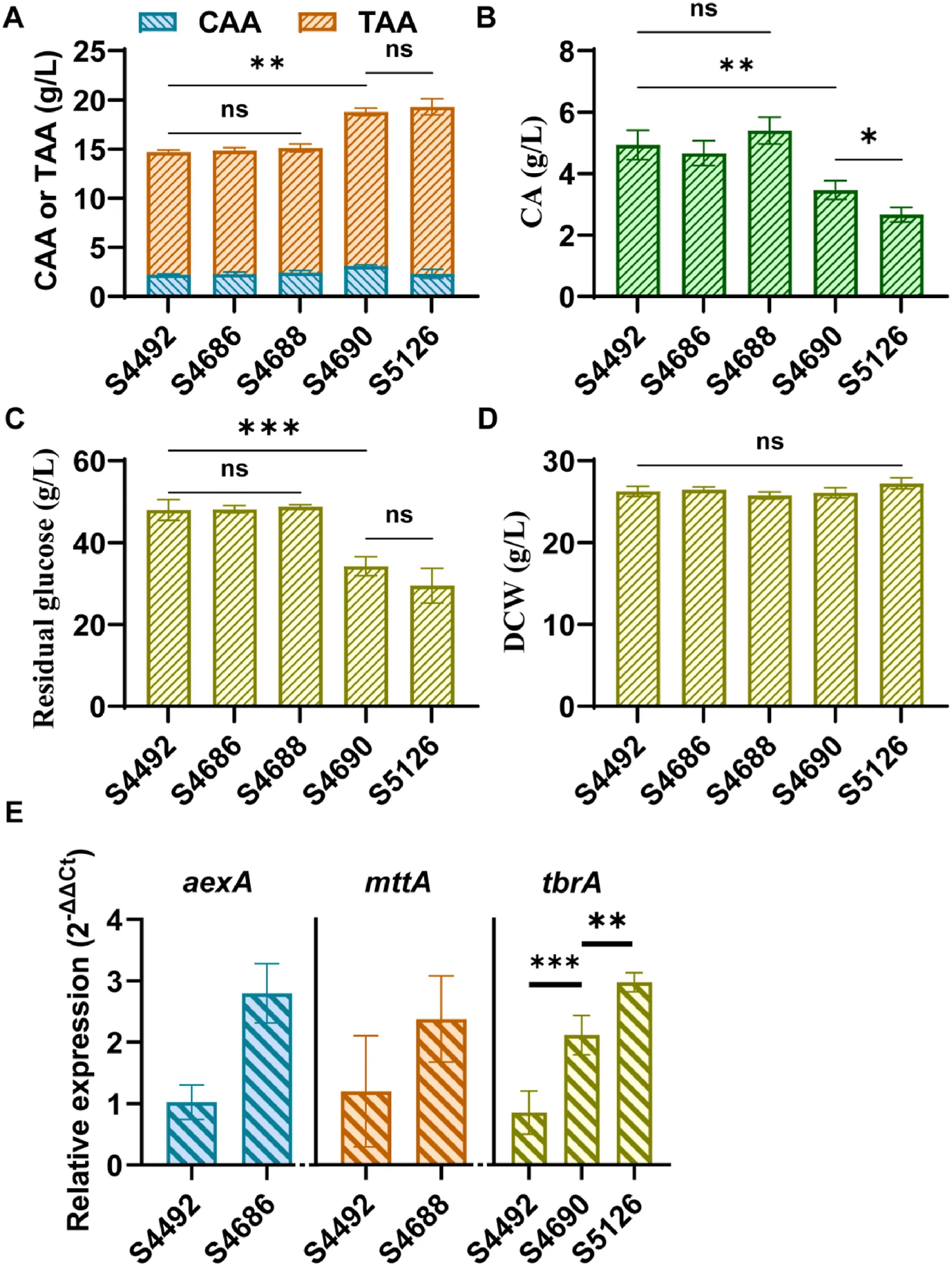
Effect of key gene copy number on AA production in 6-day shake-flask cultures. (A) AA titer, (B) CA titer, (C) residual glucose, and (D) DCW. (E) Relative transcription levels of gene analyzed by qRT-PCR. S4492 (OE-*aexA*, OE-*Ang-acoA*^M^, OE-*pk-pta*, OE-*mttA*, OE-*tbrA*), S4686 (S4492 + one additional copy of *aexA*), S4688 (S4492 + one additional copy of *mttA*), S4690 (S4492 + one additional copy of *tbrA*), and S5126 (S4690 + one additional copy of *tbrA*). Abbreviations: TAA, cis-aconitic acid; CAA, trans-aconitic acid; AA, total aconitic acid; CA, citric acid; DCW, dry cell weight.

An extra copy of *mttA* was then introduced into S4492, yielding strain S4688. Total AA reached 15.11 g/L, only a 2.8% increase over S4492. Citrate rose slightly to 5.40 g/L, and residual glucose was 48.77 g/L (Fig. 5A–C). No significant changes were observed in growth or carbon consumption (Fig. 5D). This suggests that mitochondrial CAA export is not the primary constraint with the existing copy of *mttA*.

In contrast, increasing *tbrA* dosage produced a pronounced effect. A second copy of *tbrA* was introduced into S4492, generating strain S4690. Total AA rose to 18.78 g/L, a 27.8% increase over S4492 (Fig. 5A). CAA increased to 3.11 g/L, and TAA reached 15.67 g/L. The TAA/AA ratio remained high at 83.4%. Interestingly, increasing *tbrA* dosage primarily enhanced total aconitate accumulation rather than substantially altering the TAA/CAA ratio. These results indicate that additional TbrA expression increased aconitate production without markedly changing the relative distribution of the two aconitate isomers. Citrate dropped to 3.47 g/L, a decrease of 29.6% (Fig. 5B). Residual glucose fell to 34.23 g/L (Fig. 5C). Encouraged by this result, a third copy of *tbrA* was integrated into S5120, this strain is a derivative of S4690, generating strain S5126. This strain produced 19.30 g/L total AA, a further increase of 2.8% (Fig. 5A). The TAA fraction reached 87.9%. Citrate decreased further to 2.67 g/L, a reduction of 23.1% relative to S5120 (Fig. 5B). Residual glucose dropped to 29.50 g/L (Fig. 5C). Biomass remained comparable across all strains in this series, ranging from 25.8 to 27.2 g/L (Fig. 5D).

RT-qPCR confirmed that the introduced gene copies were actively transcribed in all strains (Fig. 5E). Multi-copy integrations were detected in the respective transformants, verifying successful expression at the transcriptional level.

The gene-dosage results demonstrate a clear pattern. Additional copies of *aexA* or *mttA* did not significantly improve aconitate production. In contrast, increasing *tbrA* copy number further raised total aconitate titer while reducing citrate accumulation and residual glucose. This differential response to gene dosage identifies the CAA-to-TAA isomerization step as a major flux-controlling step under the tested conditions. The progressive decrease in citrate that accompanies higher *tbrA* transcription suggests that enhancing isomerization pulls carbon from the TCA cycle toward TAA. The small gain from the third *tbrA* copy implies that the benefit of further dosage increase is diminishing. This may reflect saturation of isomerization capacity or the emergence of other constraints.

### 3.5 Medium optimization improves aconitate production by balancing carbon and nitrogen availability and stabilizing pH

The initial fermentation medium was adapted from a published formulation with minor modifications [25]. Carbon source, organic nitrogen level, and pH buffer were selected for optimization because they are key determinants of carbon flux, biomass formation, and acid secretion. All optimization experiments were performed with strain S5126, the final engineered strain carrying multicopy *tbrA*.

Carbon source was evaluated first. The same mass concentrations of glucose and sucrose (100 g/L each) were compared. Glucose supported markedly higher aconitate production than sucrose. The total AA titer reached 18.78 g/L on glucose, compared with 12.12 g/L on sucrose (Fig. S2A). Glucose was therefore retained as the carbon source for all subsequent experiments. Organic nitrogen supplementation was assessed next. The basal medium already contained 2 g/L (NH_4_)_2_SO_4_ as an inorganic nitrogen source. Tryptone, yeast extract, and corn steep liquor powder (CSLP) were individually added at 20 g/L to evaluate their effect. A control without organic nitrogen supplementation was included. Among the tested supplements, yeast extract gave the highest total AA titer (20.83 g/L). Tryptone produced 18.91 g/L, and CSLP gave only 13.88 g/L, which was lower than the control (Fig. S2B). These results indicated that yeast extract provided the most favorable nitrogen composition among the tested supplements for aconitate production. To determine the optimal yeast extract concentration, levels of 0, 5, 10, and 20 g/L were compared. The highest total AA titer (22.53 g/L) was achieved at 5 g/L (Fig. S2C). Increasing the yeast extract concentration to 10 g/L or 20 g/L reduced aconitate production, even though biomass continued to increase. This inverse relationship suggests that excessive organic nitrogen shifted carbon allocation toward growth at the expense of product formation. Finally, pH stabilization strategies were evaluated (Fig. S2D). Three conditions were compared under otherwise identical medium composition: 1 mol/L MES buffer, no pH regulator, and 80 g/L CaCO_3_. MES buffer yielded the highest total AA titer (23.18 g/L). Without any pH stabilizer, the titer dropped to 15.37 g/L. CaCO_3_, a commonly used neutralizer in fungal organic acid fermentation, severely inhibited aconitate production in this system, yielding only 5.33 g/L. The poor performance with CaCO_3_ may reflect altered metal ion availability or osmotic effects that are detrimental to this engineered strain.

Based on these results, the optimized shake-flask medium was defined as the original medium (Section 2.1) supplemented with 5 g/L yeast extract—the key difference from the basal formulation identified through systematic screening. This optimized formulation increased total AA production from 18.78 g/L to 23.18 g/L, an improvement of 23.4% over the initial medium (Fig. 6). This optimized medium provided a consistent basis for the bioreactor scale-up experiments described in the following section.

**Fig. 6:**
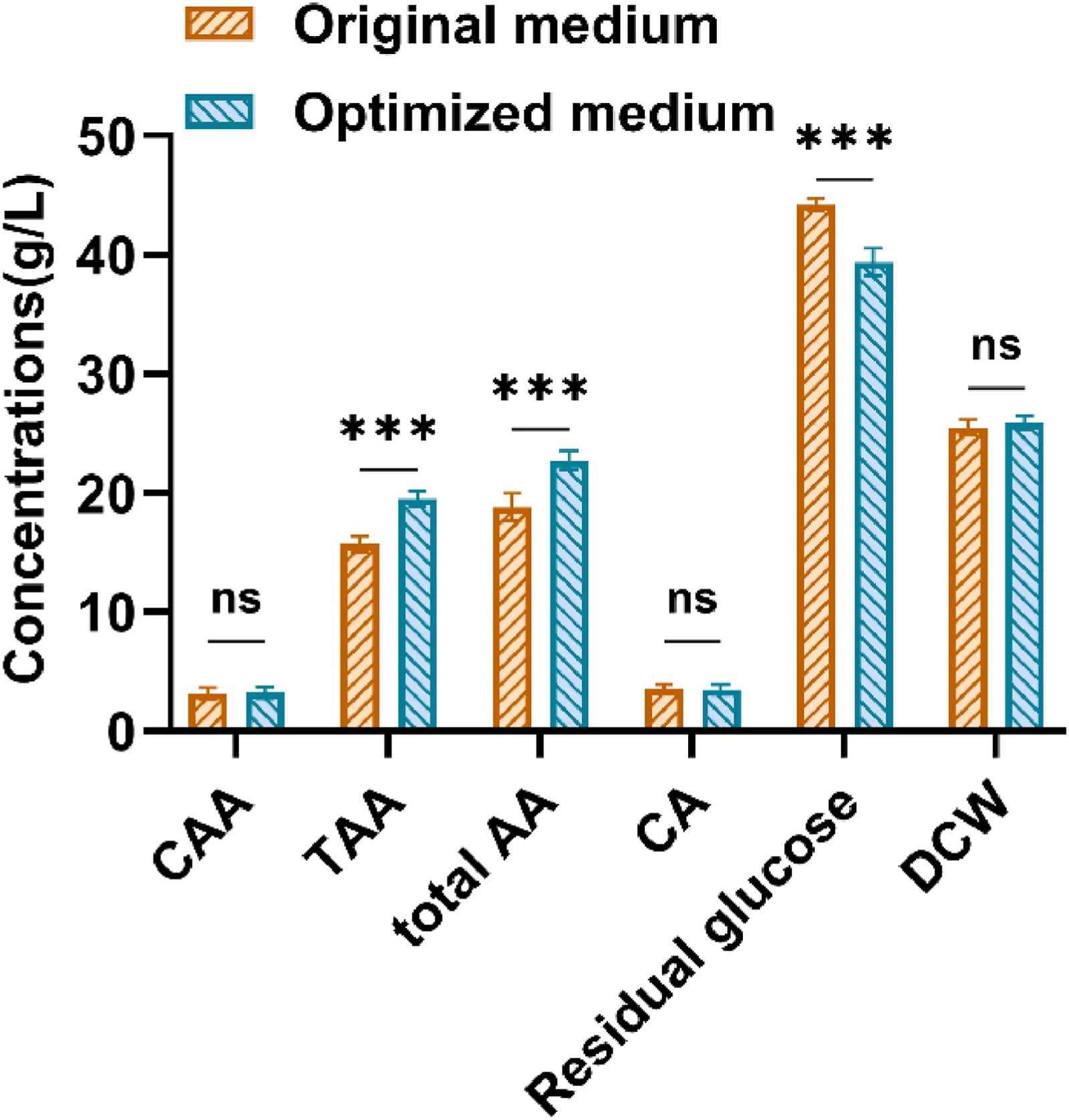
Comparison of shake-flask fermentation performance before and after medium optimization by strain S5126.

### 3.6 Bioreactor optimization and strain validation

Bioreactor conditions were optimized using the final engineered strain S5126. Agitation speed, aeration rate, and pH control strategy were examined in sequence because each influences oxygen supply and acid secretion in submerged fermentation.

Agitation speed was evaluated first at 150, 250, and 350 rpm. After 144 h, the highest total AA titer was obtained at 250 rpm (27.06 g/L), compared with 22.41 g/L at 150 rpm and 24.42 g/L at 350 rpm (Fig. S3A). Glucose was exhausted by 120 h at 250 and 350 rpm, but residual glucose remained at 150 rpm (Fig. S3B). Based on these results, 250 rpm was selected. With agitation fixed at 250 rpm, aeration was tested at 1, 2, and 3 vvm. Total AA at 144 h reached 27.06 g/L at 1 vvm, 32.67 g/L at 2 vvm, and 28.00 g/L at 3 vvm (Fig. S3C). Glucose was exhausted by 120 h under all three rates (Fig. S3D). The highest titer was achieved at 2 vvm, which was therefore selected. Using 250 rpm and 2 vvm, two pH control strategies were compared: MES buffer (1 mol/L) and automatic feeding of 4 mol/L NaOH to maintain pH 6.5. Total AA reached 32.47 g/L with MES and 34.62 g/L with NaOH (Fig. S3E). Glucose was exhausted by 120 h under both conditions, and biomass was comparable (Fig. S3F). NaOH feeding was selected for its operational simplicity. The optimized bioreactor conditions were thus: 250 rpm, 2 vvm, 35 °C, and pH 6.5 maintained by NaOH.

Under these conditions, the parental strain S1075 and the engineered strain S5126 were compared (Fig. 7). After 144 h, S1075 produced only 0.88 g/L total AA, with citrate dominating at 6.03 g/L and residual glucose remaining at 65.23 g/L. In contrast, S5126 accumulated 34.30 g/L total AA, TAA accounted for over 90% of the total, citrate dropped to 3.50 g/L, and glucose was fully consumed. The mass yield of total AA on consumed glucose reached 0.34 g/g. Time-course analysis (Fig. S3A–D) revealed distinct growth phases under all agitation and aeration conditions. A lag phase was observed from 0 to 24 h, followed by an exponential phase between 24 and 48 h, after which biomass gradually entered the stationary phase. Notably, total AA continued to accumulate until 144 h, well after biomass reached a plateau, indicating that product formation was largely non-growth associated. Overall, the engineered strain achieved an approximately 39-fold increase in aconitate titer relative to the parental strain while redirecting carbon away from citrate.

**Fig. 7:**
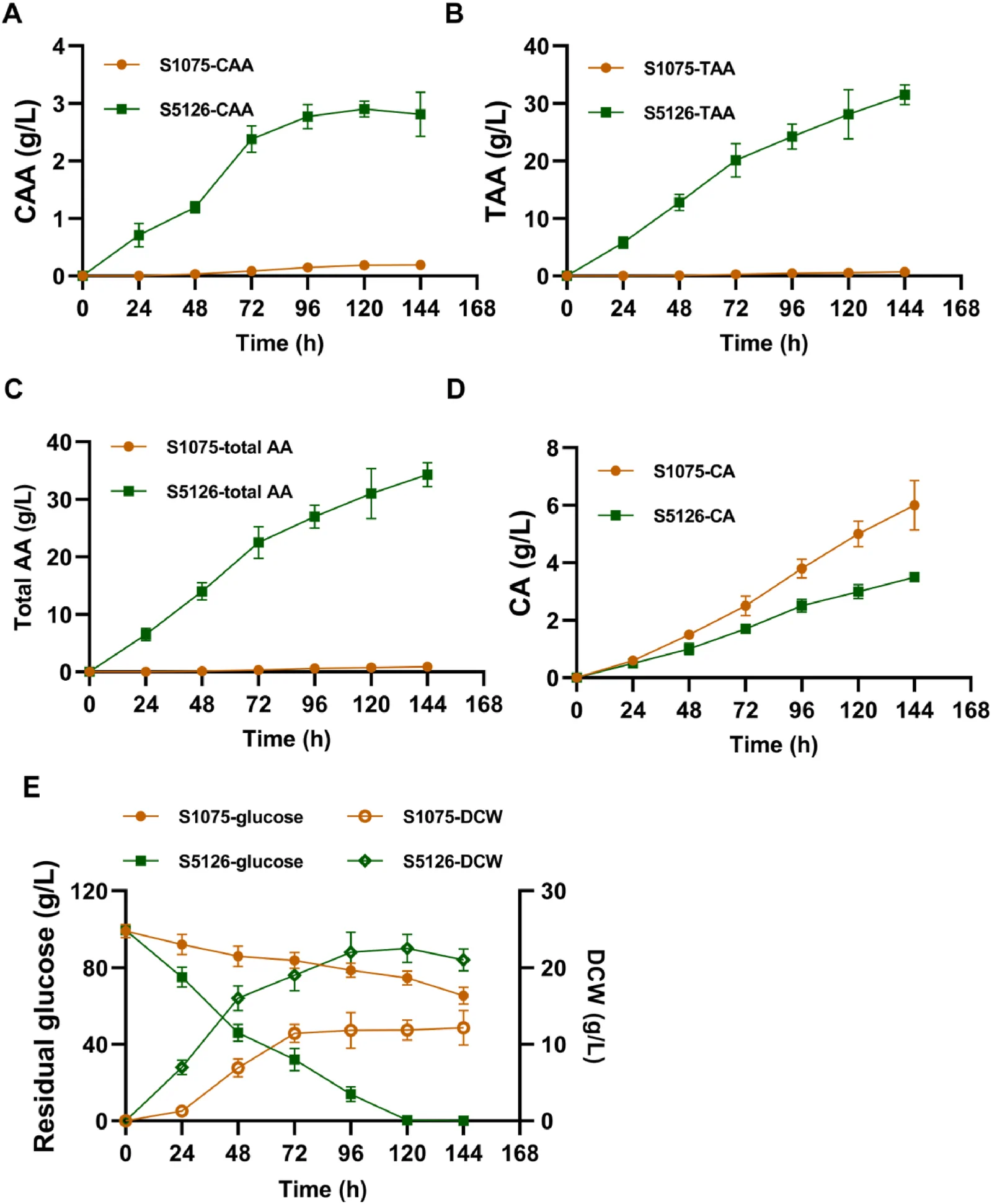
Comparison of the parental strain S1075 and the engineered strain S5126 in 5-L fermenters over 6 days. (A) CAA titer, (B) TAA titer, (C) AA titer, (D) CA titer, (E) residual sugar and DCW. Abbreviations: TAA, cis-aconitic acid; CAA, trans-aconitic acid; AA, total aconitic acid; CA, citric acid; DCW, dry cell weight.

To systematically evaluate the relationship between TAA production and biomass accumulation across different scales, we calculated the specific productivity (total AA/DCW) for all strains and conditions (Table S3). At the shake-flask level, the AA/DCW ratio increased progressively from 0.04 g/g in the parental strain S1075 to 0.71 g/g in the final engineered strain S5126, representing a ∼19-fold improvement. This increase was primarily driven by the push-pull modules, with the most substantial gain (from 0.11 to 0.56 g/g) occurring upon the introduction of MttA and TbrA, which redirected mitochondrial CAA to cytosolic TAA. Medium optimization further elevated this ratio to 0.88 g/g, indicating that nutrient adjustment enhanced cellular efficiency rather than merely increasing biomass. At the bioreactor scale, a distinct pattern emerged. While DCW in the 5-L fermenter (17–22 g/L) was consistently lower than that in shake flasks (25–27 g/L) for strain S5126, the AA/DCW ratio reached as high as 1.97 g/g under optimized conditions (250 rpm, 2 vvm, NaOH-based pH control). This value represents a ∼2.2-fold improvement over the optimized shake-flask ratio (0.88 g/g) and a ∼50-fold improvement over the parental strain in bioreactor (0.05 g/g). Notably, the highest DCW (19.67 g/L) was observed at 150 rpm, but this condition yielded the lowest AA/DCW ratio (1.14 g/g) among the tested agitation speeds, whereas the highest AA/DCW ratio (1.55 g/g) was achieved at 250 rpm with lower DCW (17.4 g/L). This inverse relationship suggests that TAA production in the bioreactor is predominantly non-growth-associated under high oxygen and controlled pH conditions. The substantial increase in specific productivity, coupled with the decrease in biomass, implies that bioreactor optimization primarily enhances the metabolic capacity per unit cell mass rather than promoting cell proliferation. This finding has practical implications for process scale-up: fed-batch or two-stage fermentation strategies (growth phase followed by production phase) could further leverage this non-growth-associated production mode to maximize TAA titer and yield.

## 4 Discussion

In this study, we systematically engineered *A. niger* into a cell factory dedicated to TAA biosynthesis. The final strain S5126 produced 34.30 g/L total aconitate in a 5-L bioreactor, with TAA accounting for over 90% of the total. This represents an approximately 39-fold improvement over the parental strain S1075, which produced only 0.88 g/L. Below we discuss the contributions and limitations of this work in the context of two recent landmark studies on microbial TAA production: Geng et al. (2023) [10] in *A. terreus* and Guo et al. (2026) in *E. coli* [14].

Citrate serves as the immediate precursor to aconitate in the TCA cycle. While it remains a bulk chemical with mature global markets, the industry is actively seeking higher-value applications for this carbon pool. Consequently, redirecting the massive carbon flux that *A. niger* naturally channels toward citrate into downstream derivatives, such as TAA, holds both significant scientific merit and economic potential. The present study occupies a complementary position within this emerging landscape. Our goal is to capitalize on the unparalleled industrial maturity and safety profile of *A. niger* to establish a robust, scalable platform for the sustainable biosynthesis of TAA.

We acknowledge that *A. terreus* and *A. pseudoterreus* are natural itaconic acid producers with carbon flux already directed toward cis-aconitate, and that *cadA* deletion readily enables aconitate accumulation [10], [11]. However, *A. niger* exhibits two highly favorable characteristics. First, *A. niger* is a GRAS organism with over half a century of safe industrial use [15], [16], [17], whereas *A. terreus* has been associated with concerns over slow growth, morphological complexity, and potential mycotoxin production [10]. Second, *A. niger* possesses established industrial-scale fermentation infrastructure with citrate production exceeding 200 g/L. Notably, this very capacity for citrate overproduction is achieved through restricted conversion of citrate to downstream TCA intermediates—the aconitase reaction strongly favors citrate under physiological conditions, and the host's regulatory network reinforces this block. Thus, engineering *A. niger* for TAA production is not a matter of tapping into an existing aconitate pathway, but rather of reopening a flux node that the native system has evolved to constrain. Encouragingly, despite this initial carbon-flux disadvantage, our shake-flask performance (23.18 g/L) is comparable to that of engineered *A. terreus* before process intensification (approximately 20 g/L) [10], and the current 5-L bioreactor titer of 34.30 g/L in batch mode—obtained without glucose feeding—indicates substantial room for further improvement. This work demonstrates that *A. niger* can be redirected from citrate overproduction to efficient TAA biosynthesis, thereby expanding the product portfolio of this industrial workhorse and adding a biosafety-certified, industrially proven eukaryotic chassis to the growing TAA production landscape.

A central finding of this study is that downstream pull interventions exhibited stronger individual effects than upstream push interventions under the tested conditions. We introduced three pull-oriented modules in sequence, and each produced substantial gains. Heterologous expression of the aconitate exporter AexA increased total aconitate 3.7-fold. Introduction of the mitochondrial CAA transporter MttA further increased the titer 2.3-fold. Heterologous expression of the aconitate isomerase TbrA produced the largest single-step improvement of 2.7-fold, and a second copy of *tbrA* contributed an additional 27.8% gain. Among the tested modules, TbrA exhibited the strongest effect on aconitate accumulation. An interesting observation in this study was that increasing TbrA dosage substantially enhanced total aconitate production, whereas the extracellular TAA/CAA ratio remained relatively constant at approximately 85/15. This indicates that the major contribution of TbrA was to enhance CAA-to-TAA conversion flux rather than simply shifting the final isomer distribution. Because aconitate-Δ-isomerase catalyzes a reversible conversion between CAA and TAA, the observed TAA/CAA ratio likely represents a dynamic steady state determined by multiple factors, including CAA generation, substrate availability, isomerization kinetics, spontaneous isomerization, and metabolite transport. In our *A. niger* system, CAA is continuously generated through mitochondrial aconitase activity and transported to the cytosol via MttA before conversion by TbrA. Therefore, increasing TbrA expression accelerates CAA consumption and promotes downstream flux, which in turn facilitates continuous replenishment of the CAA pool from upstream metabolism. As a result, enhanced TbrA activity increases the total aconitate pool without causing a proportional change in the extracellular TAA/CAA ratio. In addition, spontaneous CAA-to-TAA conversion, as observed in the parental strain lacking heterologous isomerase, may further contribute to maintaining TAA as the dominant aconitate species. Thus, TbrA should be considered primarily as a pathway pull enhancer that increases CAA-to-TAA conversion flux, while the final product isomer distribution is determined by the integrated effects of isomerization kinetics, metabolite availability, spontaneous conversion, and transport processes.

In contrast, interventions designed to push carbon into the pathway were considerably less effective. We overexpressed AcoA from three different sources and targeted the enzyme to two subcellular localizations. The best result was a 5.7% improvement. Co-expression of PK-PTA to augment acetyl-CoA supply provided only an 8.7% increase. The minimal effect of aconitase overexpression is mechanistically informative. It supports the view that the aconitase reaction operates near thermodynamic equilibrium under physiological conditions and that simply increasing enzyme abundance cannot drive net conversion of citrate to CAA when the product is not removed. The modest effect of PK-PTA further suggests that precursor supply was not the primary bottleneck in this citrate-overproducing host. Collectively, these results suggest that, under the tested conditions, downstream transport and conversion steps impose stronger limitations on TAA production than upstream carbon supply enhancement. Pull modules were correspondingly more effective than push modules in overcoming these constraints. However, the quantitative contribution and potential synergistic effects of individual push and pull modules within the final integrated strain remain to be further investigated through systematic comparison of different module combinations.

This finding presents an instructive contrast with the experience of Guo et al. [14], in *E. coli*. In that prokaryotic system, push strategies were essential to achieving the high yield of 0.76 g/g. The authors eliminated six byproduct pathways and overexpressed a feedback-insensitive pyruvate carboxylase and citrate synthase. We believe this divergence reflects genuine biological differences between the two chassis rather than the inherent superiority of one strategy over the other. *E. coli* lacks subcellular compartmentalization, meaning that intermediates are not sequestered within organelles and that competing fermentative pathways readily dissipate carbon flux if not deleted. Enforcing carbon entry into the TCA cycle through push strategies is therefore both necessary and effective. In *A. niger*, citrate is already produced at high flux and stored in the mitochondria. The rate-limiting steps therefore shift downstream, becoming the transport of CAA out of the mitochondria and its subsequent trapping as TAA. Importantly, the two strategies are not mutually exclusive but mechanistically complementary. Enhanced upstream carbon supply provides greater substrate flux for downstream pull modules, and efficient product removal alleviates feedback inhibition on upstream enzymes. A logical next step would be to systematically integrate the upstream push strategies validated in *E. coli* into our *A. niger* pull chassis. In addition to carbon flux redistribution, the two systems also differ substantially in how aconitate intermediates are generated, processed, and transported, which may influence the final CAA/TAA distribution. Beyond the difference in metabolic strategies, the distinct CAA/TAA distributions between the two systems may also reflect differences in intracellular metabolite organization and transport properties. In the *E. coli* system, Adi1 was coupled with TbrB, which was originally identified as a membrane transporter involved in extracellular accumulation of TAA. Although direct substrate specificity analysis of TbrB has not been reported, its genetic characterization suggests that TbrB may preferentially facilitate TAA export. Efficient removal of newly formed TAA may contribute to maintaining a higher apparent TAA proportion in the *E. coli* system by facilitating downstream product accumulation. In contrast, the *A. niger* platform relies on a compartmentalized pathway in which CAA is generated in mitochondria, exported through MttA, and subsequently converted to TAA by cytosolic TbrA. Therefore, the accessible intracellular CAA pool for aconitate isomerase differs from that in *E. coli*, which lacks organelle compartmentalization. Moreover, AexA introduced in our study showed broader transport characteristics, as its overexpression increased extracellular accumulation of both aconitate and citrate. Thus, differences in transporter properties, intracellular CAA availability, and compartmental organization may collectively contribute to the different CAA/TAA distributions observed between the two hosts.

A phenomenon observed in both this study and that of Geng et al. [10], merits particular attention. In *A. terreus* ΔcadA, the TAA fraction increased progressively during fermentation, reaching approximately 90% at 120 h. This was attributed to the spontaneous isomerization of CAA to TAA. The authors noted that this observation was essential because it suggested that TAA is easier to produce than CAA. In our study, the parental strain S1075 also accumulated TAA as the dominant aconitate species, accounting for 75.2% of the total, even though it lacked any heterologous isomerase. This cross-species and cross-condition reproducibility is significant. It suggests that spontaneous CAA-to-TAA isomerization may represent common behavior under cultivation conditions. The practical implication is clear: strain engineering efforts can focus on building efficient export and enzymatic trapping mechanisms rather than on creating isomerization activity from scratch. The final strain achieved over 90% TAA purity. This is consistent with the negligible CAA accumulation reported by Guo et al. [14], in their *E. coli* system. Together, these observations suggest that TAA is the predominant isomer under the cultivation conditions examined here and may represent a favorable terminal product for industrial production. From a processing standpoint, Geng et al. [10], showed that residual CAA can be converted to TAA through a post-fermentation heating step. Our study achieves comparable purity directly during fermentation through TbrA overexpression, which simplifies the overall process flow.

The parallel multi-copy integration experiment with *aexA*, *mttA*, and *tbrA* provides a useful empirical approach for identifying potential rate-limiting steps in a multi-enzyme pathway. Only additional copies of *tbrA* conferred substantial titer improvement, with a second copy yielding a 27.8% gain. Extra copies of *aexA* and *mttA* produced negligible effects. This differential response pattern can provide empirical evidence that the isomerization step exerts strong flux control under the tested conditions. The approach may prove useful for engineering other TCA cycle derivatives where flux control is distributed across several steps. The diminishing return from the third *tbrA* copy is likewise informative. The gain was merely 2.8%, suggesting that the isomerization step is approaching saturation under the current expression regime. This inference is consistent with the observation of Guo et al. [14], that a single copy of *adi1* sufficed for near-complete CAA conversion in *E. coli*. Further enhancement of this node will likely require enzyme engineering. Improving the kcat/Km toward CAA or alleviating potential product inhibition are two possible directions.

Several aspects of this study remain to be further improved. The final titer of 34.30 g/L has not yet reached the 60 g/L reported by Geng et al. [10], in *A. terreus*. However, our shake-flask titer of 23.18 g/L is comparable to the approximately 20 g/L reported for the engineered *A. terreus* strain before fed-batch intensification, and the current 5-L batch titer was obtained without glucose feeding, suggesting that fed-batch optimization and process scale-up could further narrow this gap. The yield of 0.34 g/g glucose also leaves room for improvement relative to the 0.76 g/g achieved by Guo et al. [14], in *E. coli*. Carbon loss occurs mainly through citrate co-secretion. The engineered strain still secretes 3.5 to 6 g/L citrate, and the concomitant increase in aconitate and citrate secretion upon AexA introduction suggests that AexA may transport citrate in addition to aconitate. The potential endogenous transporter candidates identified through genome analysis provide valuable targets for future functional characterization. Screening and engineering aconitate transporters with higher substrate specificity may help improve selective aconitate export and reduce citrate co-secretion. At the isomerization step, the third copy of *tbrA* yielded only a 2.8% gain, indicating that isomerization capacity is approaching saturation; enzyme engineering of TbrA to improve catalytic efficiency or relieve product inhibition will likely be key to further progress. The time-course data also indicated that biomass plateaued after approximately 96 h, whereas total AA continued to increase until the end of fermentation. This suggests that TAA formation was largely dissociated from cell growth under the tested conditions. Since agitation and aeration were kept constant in each run, future work could explore two-stage control strategies based on these growth kinetics to further improve production. Regarding process validation, this study has been conducted only at the 5-L scale in batch mode, and the morphological complexity and shear sensitivity of filamentous fungi present engineering challenges for industrial scale-up. On the positive side, *A. niger* exhibits favorable tolerance to organic acid stress, as 34.30 g/L TAA did not cause apparent growth inhibition, in contrast to the speculation of Guo et al. [14], that their *E. coli* system might be approaching TAA tolerance limits. This inherent tolerance provides a sound basis for further intensification under fed-batch or low-pH regimes. Future work may proceed along several directions, including integrating the upstream push strategies validated in *E. coli*, deploying an aconitate transporter with higher substrate specificity, performing enzyme engineering on TbrA, and advancing process scale-up.

In summary, this work establishes *A. niger* as a viable GRAS chassis for TAA biosynthesis. This work demonstrates that a compartmentalized metabolic engineering strategy incorporating mitochondrial CAA transport and downstream TAA formation can effectively redirect carbon flux from citrate toward TAA production in *A. niger*. This strategy sequentially combines extracellular aconitate export, mitochondrial CAA transport, and reversible cytosolic isomerization. In this particular chassis, downstream pull interventions exerted greater flux control than upstream push interventions, with TbrA-catalyzed isomerization representing a major flux-controlling step based on its higher response to gene dosage. A broader insight emerges when this work is considered alongside the studies of Geng et al. [10], and Guo et al. [14]. The optimal balance between push and pull strategies may well be chassis-dependent. This complementary framework can guide future efforts to maximize TAA titer, yield, and productivity in industrially relevant microbial platforms. Finally, this study exemplifies a general strategy for expanding the product portfolio of established industrial fermentation processes by reprogramming a commodity chemical producer into a producer of a higher-value downstream derivative.

## CRediT authorship contribution statement

**Song Wang:** Writing – review & editing, Writing – original draft, Validation, Methodology, Investigation, Data curation. **Jiaying He:** Validation, Data curation. **Wenhao Li:** Validation, Data curation. **Chenlu Cui:** Validation, Data curation. **Jiao Liu:** Writing – review & editing, Writing – original draft, Investigation. **Hao Liu:** Writing – review & editing, Resources, Project administration, Funding acquisition, Conceptualization.

## Declaration of generative AI and AI-assisted technologies in the writing process

AI-assisted editing (DeepSeek) was applied to enhance readability. All AI outputs were manually validated. No AI was used in data analysis or conclusion formulation.

## Declaration of competing interest

The authors declare that they have no known competing financial interests or personal relationships that could have appeared to influence the work reported in this paper.
