# Supplementary material for: Systematic metabolic engineering for trans-aconitic acid production in Aspergillus niger
Source: Synth Syst Biotechnol. 2026 Nov 2;19:93–104. doi: 10.1016/j.synbio.2026.10.007 (PMC13755589; doi:10.1016/j.synbio.2026.10.007)
Supplement: Supplementary file 1 [file mmc1.docx]

**Supplementary Information**

**Systematic metabolic engineering for trans-aconitic acid production in *Aspergillus niger***

**Author names:**

Song Wang, Jiaying He, Wenhao Li, Chenlu Cui, Jiao Liu*, Hao Liu*

**Affiliations:**

Key Laboratory of Industrial Fermentation Microbiology, Ministry of Education, Tianjin Key Laboratory of Industrial Microbiology, College of Biotechnology, Tianjin University of Science and Technology, Tianjin 300457, China.

**Postal address**:

No. 9, 13th Avenue, Tianjin Economic-Technological Development Area (TEDA) Tianjin 300457, P. R. China.

***Corresponding Author.**

***E-mail* addresses:** liuj@tust.edu.cn (J. Liu), liuhao@tust.edu.cn (H. Liu).

**List of Supporting Information**

Table S1 Strains and plasmids required for this study

Table S2 Primers used in this study.

Table S3 Summary of fermentation parameters across strain construction, medium optimization, and fermenter optimization.

Fig S1. The residual glucose and dry cell weight measured by the strains overexpressing *acoA* gene.

Fig S2. The influence of fermentation medium components on AA synthesis.

Fig S3. The influence of fermentation conditions in fermenters on AA synthesis

**Table S1 Strains and plasmids required for this study**

| Strains and Plasmids | Description | Source |
| --- | --- | --- |
| Strains |  |  |
| *E. coli* JM109 | used for plasmids construction | ^15^ |
| S1075 | Δ*oahA*, Δ*cexA*, *hyg*^s^ | ^16^ |
| S4060 | S1075, OE*aexA*, *hyg*^r^ | This study |
| S4096 | S1075, OE*aexA*, *hyg*^s^ | This study |
| S4152 | S4096, OE*Ang-acoA^C^*, *hyg*^r^ | This study |
| S4154 | S4096, OE*Ang-acoA^M^*, *hyg*^r^ | This study |
| S4156 | S4096, OE*Ate*-*acoA^C^*, *hyg*^r^ | This study |
| S4158 | S4096, OE*Ate*-*acoA^M^*, *hyg*^r^ | This study |
| S4160 | S4096, OE*Sc*-*acoA^C^*, *hyg*^r^ | This study |
| S4162 | S4096, OE*Sc*-*acoA^M^*, *hyg*^r^ | This study |
| S4216 | S4154, OE*Ang-acoA^M^*, *hyg*^s^ | This study |
| S4370 | S4216, OE*pk-pta*, *hyg*^r^ | This study |
| S4373 | S4216, OE*pk-pta*, *hyg*^s^ | This study |
| S4420 | S4373, OE*mttA*, *hyg*^r^ | This study |
| S4423 | S4373, OE*mttA*, *hyg*^s^ | This study |
| S4427 | S4423, *OEadiA*, *hyg*^r^ | This study |
| S4430 | S4423, *OEtbrA*, *hyg*^r^ | This study |
| S4492 | S4423, *OEtbrA*, *hyg*^s^ | This study |
| S4686 | S4492, OE*aexA* (two-copys), *hyg*^r^ | This study |
| S4688 | S4492, OE*mttA* (two-copys), *hyg*^r^ | This study |
| S4690 | S4492, *OEtbrA* (two-copys), *hyg*^r^ | This study |
| S5120 | S4492, *OEtbrA* (two-copys), *hyg*^s^ | This study |
| S5126 | S5120, *OEtbrA* (three-copys), *hyg*^r^ | This study |
| Plasmids |  |  |
| pLH454 | *lox*P-*hph-lox*P, P*_gpdA_*, T*_trpC_*, *hyg*^r^, *kan*^r^ | ^15^ |
| pLH509 | *lox*P-*hph-lox*P, P*_pkiA_*, T*_trpC_*, *hyg*^r^, *kan*^r^ | ^16^ |
| pLH2156 | pLH454, P*_gpdA_*, *aexA*, T*_trpC_* | This study |
| pLH2184 | pLH454, P*_gpdA_*, *Ang*-*acoA^C^*, T*_trpC_* | This study |
| pLH2185 | pLH454, P*_gpdA_*, *Ang-acoA^M^*, T*_trpC_* | This study |
| pLH2186 | pLH454, P*_gpdA_*, *Ate*-*acoA^C^*, T*_trpC_* | This study |
| pLH2187 | pLH454, P*_gpdA_*, *Ate*-*acoA^M^*, T*_trpC_* | This study |
| pLH2188 | pLH454, P*_gpdA_*, *Sc*-*acoA^C^*, T*_trpC_* | This study |
| pLH2189 | pLH454, P*_gpdA_*, *Sc-acoA^M^*, T*_trpC_* | This study |
| pLH2331 | pLH509, P*_pkiA_*, *mttA*, T*_trpC_* | This study |
| pLH2076 | pLH454, P*_gpdA_*, *pta*, T*_trpC_*, P*_tpiA_*, *pk*, T*_argB_* | This study |
| pLH2342 | pLH454, P*_gpdA_*, *tbrA*, T*_trpC_* | This study |
| pLH2343 | pLH454, P*_gpdA_*, *adiA*, T*_trpC_* | This study |

hyg^r^, hygromycin B resistance; hyg^s^, hygromycin B sensitive.

**Table S2 Primers used in this study**

| primer name | | Primer sequences (5'→3') |
| --- | --- | --- |
| Amplify the *aexA* sequence | | |
| Primer 1 | | *ACATCTAAACAATGG*AATTCAATGACCCGCGACGAGCACG |
| Primer 2 | | *TCAGTAACGTTAAGT*GGATCCTCAGTAGCCGTAGACGGAGG |
| Amplify of the *acoA* sequence from *Aspergillus niger* is localized in the cytosolic | | |
| Primer 3 | | *ATAGACACATCTAAA*CAATGGAATTCATGGCCACCGTCACCGAC |
| Primer 4 | | *TGATTTCAGTAACGT*TAAGTGGATCCTTAGTTGCCAGCCTTGCGG |
| Amplify of the *acoA* sequence from *Aspergillus niger* is localized in the mitochondrion | | |
| Primer 5 | | *ATAGACACATCTAAA*CAATGGAATTCATGATTACCACAAGGCTTGCG |
| Primer 6 | | *TGATTTCAGTAACGT*TAAGTGGATCCTTAGTTGCCAGCCTTGCGG |
| Amplify of the *acoA* sequence from *Aspergillus terreus* is localized in the cytosolic | | |
| Primer 7 | | *ATAGACACATCTAAAC*AATGGAATTCATGGCCACCGTCGCCGACTCC |
| Primer 8 | | *TGATTTCAGTAACGT*TAAGTGGATCCTCAGTTGGAGGCGGCCTTGC |
| Amplify of the *acoA* sequence from *Aspergillus terreus* is localized in the mitochondrion | | |
| Primer 9 | | *CACATCTAAACAATG*GAATTCATGATTTCCACCCGCCTG |
| Primer 10 | | *TCAGTAACGTTAAGT*GGATCCTCAGTTGGAGGCGGCCTTG |
| Amplify of the *acoA* sequence from Saccharomyces cerevisiae is localized in the cytosolic | | |
| Primer 11 | | *ATAGACACATCTAAACAATG*GAATTCATGGCGACAGTCTCCAAC |
| Primer 12 | | *TGATTTCAGTAACGTTAAGT*GGATCCTTATTTCTTCTCATCGGCCTTAAT |
| Amplify of the *acoA* sequence from Saccharomyces cerevisiae is localized in the mitochondrion | | |
| Primer 13 | | *ATAGACACATCTAAACAAT*GGAATTCATGCTGTCTGCACGTTCT |
| Primer 14 | | *TGATTTCAGTAACGTTAAGT*GGATCCTTATTTCTTCTCATCGGCCTTAAT |
| Amplify the *pk* sequence | | |
| Primer 15 | | *CTCACTGGTACCTGT*ACTAGTTCTCAAGCCGTGAAACCA |
| Primer 16 | | *GTTGCGGCCGCAGCT*GGATCCACCTACAGCCATTGCGAA |
| Amplify the *pta* sequence | | |
| Primer 17 | | *TCAAGCTTCATGAATTCAGC*TCTAGAGAGACTAGTGGACTAACATTA |
| Primer 18 | | *CAGGTACCAGTGAGCTCATC*TCTAGAAGTCACGACGTTGTAAAA |
| Amplify the *mttA* sequence | | |
| Primer 19 | | *GATGGAATTCAATTC*GAGCTCATGGACTCCAAGATTCAGA |
| Primer 20 | | *TCCAGATCTCTGCAG*GGTACCTCAGTTGGGCTGAGTCAGG |
| Amplify the *tbrA* sequence | | |
| Primer 21 | | *ATAGACACATCTAAA*CAATGGAATTCATGAAGATCCCCT |
| Primer 22 | | *TTCAGTAACGTTAAG*TGGATCCTCAGGGGATGATCAGCT |
| Amplify the *adiA* sequence | | |
| Primer 23 | *TCATCCGTCAAGATG*GAATTCATGCTGCACCCCATTGAC | |
| Primer 24 | *TCCAGATCTCTGCAG*GGTACCTCAGGACAGGGAGCGGTC | |

**Table S3 Summary of fermentation parameters across strain construction, medium optimization, and fermenter optimization.**

| Strain/  Condition | Genotype | CAA (g/L) | TAA (g/L) | Total AA (g/L) | DCW (g/L) | Total AA/DCW (g/g) |
| --- | --- | --- | --- | --- | --- | --- |
| Shake-flask fermentation | | | | | | |
| S1075 | Δ*oahA*, Δ*cexA*, *hyg*^s^ | 0.15 | 0.44 | 0.58 | 15.67 | 0.04 |
| S4060 | S1075, OE*aexA*, *hyg*^r^ | 0.45 | 1.67 | 2.12 | 17.8 | 0.12 |
| S4096 | S1075, OE*aexA*, *hyg*^s^ | 0.45 | 1.64 | 2.08 | 17.8 | 0.12 |
| S4152 | S4096, OE*Ang-acoA^C^*, *hyg*^r^ | 0.41 | 1.26 | 1.67 | 20.7 | 0.08 |
| S4154 | S4096, OE*Ang-acoA^M^*, *hyg*^r^ | 0.55 | 1.69 | 2.24 | 19.5 | 0.11 |
| S4156 | S4096, OE*Ate*-*acoA^C^*, *hyg*^r^ | 0.35 | 1.28 | 1.62 | 20.4 | 0.08 |
| S4158 | S4096, OE*Ate*-*acoA^M^*, *hyg*^r^ | 0.52 | 1.54 | 2.06 | 19.6 | 0.11 |
| S4160 | S4096, OE*Sc*-*acoA^C^*, *hyg*^r^ | 0.42 | 1.34 | 1.76 | 19.3 | 0.09 |
| S4162 | S4096, OE*Sc*-*acoA^M^*, *hyg*^r^ | 0.49 | 1.62 | 2.11 | 18.9 | 0.11 |
| S4216 | S4154, OE*Ang-acoA^M^*, *hyg*^s^ | 0.49 | 1.69 | 2.18 | 19.5 | 0.11 |
| S4370 | S4216, OE*pk-pta*, *hyg*^r^ | 0.56 | 1.81 | 2.38 | 24.7 | 0.1 |
| S4373 | S4216, OE*pk-pta*, *hyg*^s^ | - | - | - | - | - |
| S4420 | S4373, OE*mttA*, *hyg*^r^ | 0.83 | 4.67 | 5.5 | 26.5 | 0.21 |
| S4423 | S4373, OE*mttA*, *hyg*^s^ | - | - | - | - | - |
| S4427 | S4423, *OEadiA*, *hyg*^r^ | 1.85 | 10.25 | 12.1 | 26.2 | 0.46 |
| S4430 | S4423, *OEtbrA*, *hyg*^r^ | 2.23 | 12.47 | 14.7 | 26.27 | 0.56 |
| S4492 | S4423, *OEtbrA*, *hyg*^s^ | 2.23 | 12.47 | 14.7 | 26.27 | 0.56 |
| S4686 | S4492, OE*aexA* (two-copys), *hyg*^r^ | 2.3 | 12.56 | 14.86 | 26.47 | 0.56 |
| S4688 | S4492, OE*mttA* (two-copys), *hyg*^r^ | 2.45 | 12.66 | 15.11 | 25.77 | 0.59 |
| S4690 | S4492, *OEtbrA* (two-copys), *hyg*^r^ | 3.11 | 15.67 | 18.78 | 26.1 | 0.72 |
| S5120 | S4492, *OEtbrA* (two-copys), *hyg*^s^ | - | - | - | - | - |
| S5126 | S5120, *OEtbrA* (three-copys), *hyg*^r^ | 2.32 | 16.97 | 19.3 | 27.23 | 0.71 |
| Medium optimization | | | | | | |
| Original medium | - | 3.11 | 15.67 | 18.78 | 25.48 | 0.74 |
| Optimized medium | - | 3.2 | 19.48 | 22.68 | 25.88 | 0.88 |
| Fermenter optimization | | | | | | |
| 150 rpm | - | 2.8 | 19.61 | 22.41 | 19.67 | 1.14 |
| 250 rpm | - | 3.81 | 23.25 | 27.06 | 17.43 | 1.55 |
| 350 rpm | - | 3.21 | 21.21 | 24.42 | 19 | 1.28 |
| 1 vvm | - | 3.7 | 23.36 | 27.06 | 17.06 | 1.59 |
| 2 vvm | - | 4.29 | 28.39 | 32.67 | 21.51 | 1.52 |
| 3 vvm | - | 3.75 | 24.25 | 28 | 19.6 | 1.43 |
| MES | - | 4.3 | 28.3 | 32.6 | 21 | 1.55 |
| NaOH | - | 4.5 | 30.1 | 34.6 | 20 | 1.73 |
| Fermenter fermentation comparison | | | | | | |
| S1075 | - | 0.19 | 0.69 | 0.88 | 18.33 | 0.05 |
| S5126 | - | 2.81 | 31.49 | 34.3 | 17.43 | 1.97 |

**Fig S1**. **The residual glucose and dry cell weight measured by the strains overexpressing *acoA* gene**. (A): The residual glucose and dry cell weight measured by the strains overexpressing *acoA* localized in the cytoplasm in shake flasks for 6 days. (B): The residual glucose and dry cell weight measured by the strains overexpressing *acoA* localized in the mitochondria in shake flasks for 6 days. Data represent mean ± SD (n = 3 biological replicates). Statistical significance was determined using one-way ANOVA (A) or two-way ANOVA (B–D) (ns >0.05, *p < 0.05, **p < 0.01, ***p < 0.001, ****p < 0.0001).

**Fig S2. The influence of fermentation medium components on AA synthesis.** (A): Effect of carbon sources on the synthesis of AA. (B): Effect of nitrogen sources on the synthesis of AA. (C): Effect of nitrogen source concentration on the synthesis of AA. (D): Effect of pH stabilizers on the synthesis of AA. Data represent mean ± SD (n = 3 biological replicates). Statistical significance was determined using one-way ANOVA (A) or two-way ANOVA (B–D) (ns >0.05, *p < 0.05, **p < 0.01, ***p < 0.001, ****p < 0.0001).

**Fig S3. The influence of fermentation conditions in fermenters on AA synthesis.** (A): AA titer under different agitation speed in fermenters. (B): The residual glucose and dry cell weight under different agitation speed in fermenters. (C): AA titer under different aeration in fermenters. (D): The residual glucose and dry cell weight under different aeration in fermenters. (E): AA titer under different pH control strategies in fermenters. (F): The residual glucose and dry cell weight under different pH control strategies in fermenters. Data represent mean ± SD (n = 3 biological replicates). Statistical significance was determined using one-way ANOVA (A) or two-way ANOVA (B–D) (ns >0.05, *p < 0.05, **p < 0.01, ***p < 0.001, ****p < 0.0001).
